# Supplementary material for: Private Doctors’ Perspective towards “Patient First” in TB Diagnostic Cascade, Hisar, India
Source: Diagnostics (Basel). 2024 May 31;14(11):1164. doi: 10.3390/diagnostics14111164 (PMC11171542; doi:10.3390/diagnostics14111164)
Supplement: Supplementary file 1 [file diagnostics-14-01164-s001.zip › diagnostics-2990028-supplementary.pdf]

Supplement Table S1 Results of specimens from private facility completing diagnostic algorithm for Rifampicin Sensitive and Rifampicin Resistant Tuberculosis

A total of 349 specimens with Rifampicin Sensitive (RS) TB and 25 specimens with Rifampicin Resistant (RR) TB, completed the diagnostic algorithm (Table). Out of 349 specimens with RS, 291 were Isoniazid and Rifampicin sensitive in FL-LPA tests and 14 specimens were resistant to Rifampicin. Following SL-LPA tests for 16 specimens, only two specimens were resistant to FQ/ second-line injectable drugs. Liquid culture was done for specimens with FL-LPA smear negative and those with resistant to any drugs. Out of 68 tests done, 18 were culture positive and one specimen each were resistant to moxifloxacin and pyrazinamide. Only one specimen did not complete the algorithm for insufficient specimen quantity. The RR specimens also followed the diagnostic algorithm. Out of 25 specimens, 4 specimens were resistant to Rifampicin and 4 to Isoniazid. Five specimens which were smear negative were also culture negative and thus completed the algorithm. The SL-LPA tests was performed for 20 specimens out of which 5 were resistant to FQ/ second-line injectable drugs. Liquid culture test was performed for all 25 specimens and 11 showed positive results. The culture results showed one specimen resistant to moxifloxacin and 4 to pyrazinamide.

Table S1 – Results of specimens from private facility completing diagnostic algorithm for Rifampicin Sensitive and Rifampicin Resistant Tuberculosis

		Rifampicin Sensitive (RS) TB Diagnostic Algorithm		Rifampicin Resistant (RR) TB Diagnostic Algorithm	
**sl no**	**Particulars**	Number	Percentage	Number	Percentage
1	Number of RS TB Patients diagnosed at Xpert Lab	349			
2	Number of RR-TB patients diagnosed at Xpert Lab			25	100%
3	Number of samples reached the reference lab	349*	100%	25	100%
4	FL-LPA done	294	84%	20	80%
	FL-LPA done after culture positive	13	13%	0	0
	FL-LPA not done	1	<1%	0	0
i	H and R Sensitive	291	99%	12	60%
ii	H Resistant and R Sensitive	14	4.7%	0	0
iii	R Resistant only	0	0	4	20
Iv	H and R Resistant	2	<1%	4	20
iv	No results	0	0	0	0
5	SLLPA done	16	100%	20	100%
6	SL-LPA not done	0	0	0	0
i	FQ and SLI Sensitive	14	87%	15	75
ii	FQ and/ or SLI Resistant	2	12.5%	5	25
iii	No results	0		0	0
7	Culture done	68	100%	25	100%
	Culture positive	18	24%	11	44%
8	Result for LC-DST (Moxi, PZA,Lzd)	Moxi (1/18) PZA (1/18).		Moxi (1/11) PZA (4/11).	

9	Number of RS Patients completing the diagnostic algorithm	349	100%	25	100%

** One Rifampicin Indeterminate specimen included. Abbrivations; Moxi – moxifloxacin, Lzd- Linezolid, PZA – pyrazinamide, H – Isoniazid, R – Rifampicin, FL LPA – First Line LPA, SL LPA – Second Line LPA, FQ – Fluroquinolone. RS – Rifampicin Sensitive. SLI – Second Line Injectable. LC – DST – Liquid Culture Drug susceptibility test.*

**Reasons not listed. Moxi – moxifloxacin, Lzd- Linezolid, PZA – pyrazinamide, H – Isoniazid, R – Rifampicin, FL-LPA – First Line LPA, SL-LPA – Second Line LPA, FQ – Fluroquinolone. RS – Rifampicin Sensitive. SLI – Second Line Injectable. LC – DST – Liquid Culture Drug susceptibility test.*
